# Ultrafast optical-ultrasonic system and miniaturized catheter for imaging and characterizing atherosclerotic plaques *in vivo*

**DOI:** 10.1038/srep18406

**Published:** 2015-12-18

**Authors:** Jiawen Li, Teng Ma, Dilbahar Mohar, Earl Steward, Mingyue Yu, Zhonglie Piao, Youmin He, K. Kirk Shung, Qifa Zhou, Pranav M. Patel, Zhongping Chen

**Affiliations:** 1 Beckman Laser Institute, University of California, Irvine, 1002 Health Sciences Rd. Irvine, CA 92617, USA; 2 Department of Biomedical Engineering, University of California, Irvine, Irvine, CA, 92697-2700, USA; 3 NIH Ultrasonic Transducer Resource Center, University of Southern California, Los Angeles, CA 90089, USA; 4 School of Medicine, University of California, Irvine, 101 The City Drive South, Orange, CA, 92868, USA

## Abstract

Atherosclerotic coronary artery disease (CAD) is the number one cause of death worldwide. The majority of CAD-induced deaths are due to the rupture of vulnerable plaques. Accurate assessment of plaques is crucial to optimize treatment and prevent death in patients with CAD. Current diagnostic techniques are often limited by either spatial resolution or penetration depth. Several studies have proved that the combined use of optical and ultrasonic imaging techniques increase diagnostic accuracy of vulnerable plaques. Here, we introduce an ultrafast optical-ultrasonic dual-modality imaging system and flexible miniaturized catheter, which enables the translation of this technology into clinical practice. This system can perform simultaneous optical coherence tomography (OCT)-intravascular ultrasound (IVUS) imaging at 72 frames per second safely *in vivo*, i.e., visualizing a 72 mm-long artery in 4 seconds. Results obtained in atherosclerotic rabbits *in vivo* and human coronary artery segments show that this ultrafast technique can rapidly provide volumetric mapping of plaques and clearly identify vulnerable plaques. By providing ultrafast imaging of arteries with high resolution and deep penetration depth simultaneously, this hybrid IVUS-OCT technology opens new and safe opportunities to evaluate in real-time the risk posed by plaques, detect vulnerable plaques, and optimize treatment decisions.

Nearly one-half of all deaths in Europe and the United States are caused by atherosclerosis^1^,^2^. Atherosclerotic plaque is susceptible to chronic occlusion or to acute disruption and subsequent complications which lead to death. Unfortunately, tremendous technical challenges hinder the identification of such plaque sub-types and the prevention of these deaths. These challenges are related to the fact that the majority of atherosclerotic plaques are usually benign while a minority may become vulnerable plaques and unstable. These vulnerable plaques are prone to cause acute coronary syndrome (ACS), such as acute myocardial infarction and stroke. As a result, accurate identification and selective treatment of such culprit vulnerable plaques may be essential to develop methods for effective treatment and management of atherosclerosis^3^,^4^,^5^.

One predominant type of vulnerable plaque at greatest risk of causing atherosclerotic complications is thin-cap fibroatheroma (TCFA). TCFA is characterized by a thin fibrous cap (<60 μm) overlaying on a large necrotic pool within the vessel wall. In order to accurately detect this pivotal type of vulnerable plaque and possibly guide treatment, a tool that can examine those two characteristics in a cross stection of the internal structure is necessary. Although, non-invasive imaging techniques are improving, they are not currently sensitive or specific enough to accurately identify plaque due to their limited resolution^6^,^7^,^8^.

Minimal invasive methods, such as intravascular ultrasound (IVUS) and optical coherence tomography (OCT)^9^,^10^, which enable high resolution imaging within the vessel wall, have been gradually used in clinical practice. However, their use is limited^11^,^12^ due to the intrinsic technical drawbacks when using one single technology alone. OCT provides powerful resolution capability for detecting thin fibrous caps and other plaque characteristics but lacks the imaging depth necessary to quantify plaque volume and lipid/necrotic core size. IVUS has an excellent imaging depth range but lacks the high resolution and sensitivity. Thus, these two imaging modalities provide complementary information of the two major characteristics of vulnerable plaque. IVUS can provide visualization of the whole plaque burden, while OCT can measure the fibrous cap thickness. There were several studies that demonstrated the limitation of using a separated OCT or IVUS system^11^,^15^,^16^,^17^,^18^ to charcterize plaques. These studies also demonstrated that the combined use of OCT and IVUS can significantly improve the ability to diagnose vulnerable plaques^9^,^13^,^14^,^15^,^16^.

Inspired by these conclusions and the desire to accurately identify vulnerable plaque, the integrated IVUS-OCT technology was developed^17^,^18^,^19^,^20^. However, the translation of such a technology into clinical practice was greatly hindered by the large gap between IVUS and OCT imaging speeds^21^. A speed of 30 frames per second (fps) is commonly used in a commercially available IVUS system, while commercial OCT systems usually perform at over 100 fps^22^,^23^. An integrated IVUS-OCT system can only operate at the lower speed, i.e. that of IVUS. Low speed imaging, and thus long imaging procedure time, may increase the chance of catheter-induced spasm^24^. Limitations also exist in the requirement of agents for concurrent contrast injection during imaging. Larger quantities of these agents are required with low speed imaging and so may lead to renal function disorder^25^, life-threatening cardiotoxic effects and seizures. Thus, this speed limit became a fundamental barrier for the transition of an integrated IVUS-OCT into clinical practice.

Here, we successfully addressed this challenge through the development and *in vivo* application of an ultrafast integrated IVUS-OCT imaging system and catheter. The IVUS-OCT technique provides the complementary advantages of both OCT and IVUS. This system can image at 72 fps and greatly improves the safety and efficiency of using an IVUS-OCT system. We imaged the aortas of atherosclerotic rabbits, and IVUS-OCT images of vulnerable plaques were acquired. With such hybrid multimodular imaging we were able to demonstrate two crucial characteristics: the thin cap and the large necrotic core. This technology holds great promise in detecting vulnerable plaque and optimizing treatment methods^26^ in the near future. With the development of this powerful imaging tool, we believe that our ability to manage post-procedure complications and reduce repeat revascularization procedures will also be improved.

## Results

### Ultrafast imaging system and catheter

**Figure 1:**
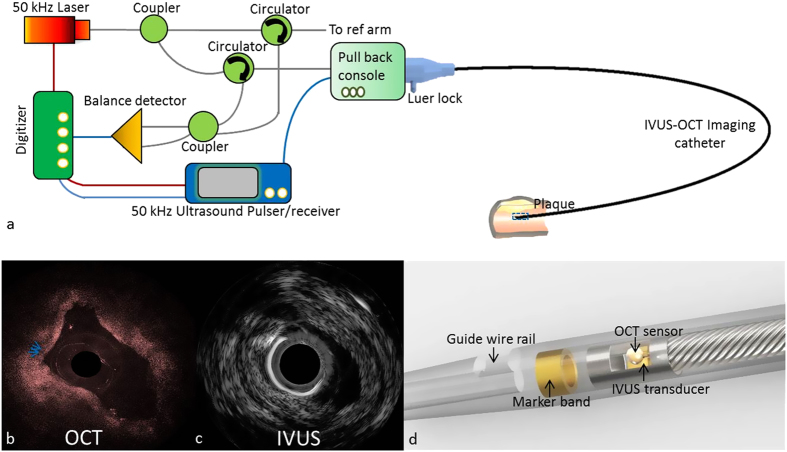
Ultrafast IVUS-OCT imaging system and catheter. (**a**) Schematic of IVUS-OCT imaging system. Gray, blue, and red lines denote the transmissions of the OCT signal through the optical fiber, the electrical signals, and the trigger signals through electrical wires, respectively. The obtained OCT (**b**) IVUS (**c**) images of the same region of interest. (**d**) A zoom-in image of the distal end of the imaging catheter.

An ultrafast IVUS-OCT imaging system (Fig. 1) was built based on a 50 kHz swept source laser and a 50 kHz-external-triggered ultrasound pulser/receiver. A 150 MHz balance detector and a 500 M samples/s two channel digitizer were used. OCT and ultrasound signal were acquired by the channel 1 and 2 of the digitizer, respectively. The A-line trigger of the swept laser was used as the trigger signal for the digitizer. The AUX port of the digitizer was connected to the external trigger-in port of the US receiver for synchronizing. A pre-trigger sampling was used for the IVUS channel in order to acquire the IVUS signal from the beginning of a pulser. The rotation was generated by an external motor and transferred to the integrated probe by a timing belt. Transmission of optical and electrical signals from the rotary part to the stationary part was achieved by an optical rotary joint (maximal speed: 10000 RPM) and a custom designed brushed electrical slip ring (maximal rotational speed: 5000 RPM). The rotational and pullback system was enclosed in an aluminum box and tested for electrical safety. The rotational and pullback speed was set at 72 rotations/s and 1.8 cm/s, respectively. A slip ring holder (with wire protection and electromagnetic shielding) was designed to secure the slip ring at the proximal end of the catheter and connected the bracket supporting the fiber rotary joint and motor. This connection was made possible by magnets. All acquired frames were transferred to the onboard memory of a graphic processing unit, which enabled enough computational speed to allow for simultaneous 72 fps processing and displaying. All processed images with the raw data were saved in real-time in an ultrafast solid state drive.

A good catheter design was also needed to achieve the goal of safe intravascular imaging *in vivo*. There were many challenges in designing an effective catheter that were overcome. These challenges included the strict size constraints of the integrated probe (so that it fit the inner diameter of human coronary arteries, 2–8 mm), the flexibility and pushability of the catheter (so that it could navigate smoothly through the human artery tree), the transparency and low toxicity of the catheter material, and the catheter’s adaptability for clinical procedures. To fit the catheter’s strict size requirements, we used a miniaturized IVUS-OCT probe design (with an outer diameter of 0.9 mm and a rigid part of 1.5 mm, see Fig. 1d). To fit the necessary mechanical requirements, transparency and low toxicity, the materials for the catheter sheath were carefully selected. Then a catheter sheath with a guide-wire rail and radiopaque marker was manufactured and used in *in vivo* experiments to fit the physician’s interventional imaging workflow (see Fig. 1d).

### Evaluation of ultrafast ultrasound sub-system

Commercially available IVUS systems usually image at 30 fps^22^,^23^. A high imaging speed was hypothesized to degrade IVUS image quality due to strong vibrations in the catheter, a high level of noise generated in the coupling process, and a short detection duration for the ultrasound transducer^19^. To quantitatively evaluate the imaging quality of our ultrasound sub-system, we tested the contrast to noise ratio (CNR) of our system in a well-controlled phantom, imaging at 25 fps, 50 fps and 72 fps. We used “wire in silicone gel” as a phantom and used the same imaging catheter throughout the test to avoid unknown variables in the animal experiments. The phantom tested showed the CNR of the ultrasound sub-system at 25 fps, 50 fps and 72 fps were 6.49 dB, 6.19 dB and 6.18 dB, respectively. There was only 0.31 dB difference between 25 fps and 72 fps, i.e., a very subtle CNR drop. This sensitivity drop can be compensated for by using our ultra-sensitive ultrasound transducer^27^.

### Ultrafast imaging rabbit artery *in vivo*

**Figure 2:**
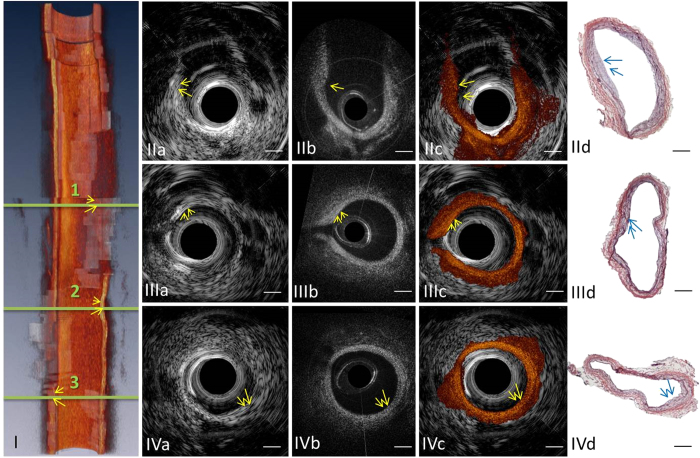
Ultrafast imaging of a rabbit abdominal aorta *in vivo*. (**I**) Three-dimensional cut-away rendering of the volumetric data set acquired with an intravascular catheter in abdominal aorta of a live rabbit. The volume comprises 288 frames of images acquired in 4 s during the injection of iohexol at a rate of 3 ml/s. Red, artery wall; semi-transparent white, lipid. Circular cross-section IVUS (**IIa**) (**IIIa**) (**IVa**) OCT (**IIb**) (**IIIb**) (**IVb**) fused IVUS-OCT **(IIc**) (**IIIc**) (**IVc**) image pairs and the corresponding H&E histology photos (**IId**) (**IIId**) (**IVd**) at locations 1, 2 and 3 denoted in (**I**). Arrows point at lipid-rich plaque regions. Scale bar: 0.5 mm. The shape of this artery changed between *in vivo* imaging and histology due to the reduced intra-lumen pressure after this artery was harvested.

To determine whether this system could provide enough mechanical stability, system robustness, handling capability and imaging quality in clinical settings at such a high speed, live healthy rabbits and atherosclerotic rabbits were imaged. Imaging at 72 fps was performed within the lumen of rabbit abdominal aortas, which are of similar caliber to human coronary arteries. A four-second flushing agent injection was applied at a speed of 3 ml/s to clear OCT images, i.e., the same amount of flushing agent as used in clinical OCT. The pull-back was achieved by a computer controlled linear stage at a rate of 1.8 cm/s. Volumetric imaging of arteries was obtained (Fig. 2I and the video in the supplementary information). According to IVUS-OCT images, plaques can be clearly visualized and characterized (Fig. 2II–IV). Regions of interest (ROIs) with intimal thickening that were observed by IVUS and OCT images (Fig. 2IIa,b, IIIa,b, Iva,b) correlated well with ROIs with pathological intimal thickening in Fig. 2IId,IIId and IVd.

### Imaging of vulnerable plaques

**Figure 3:**
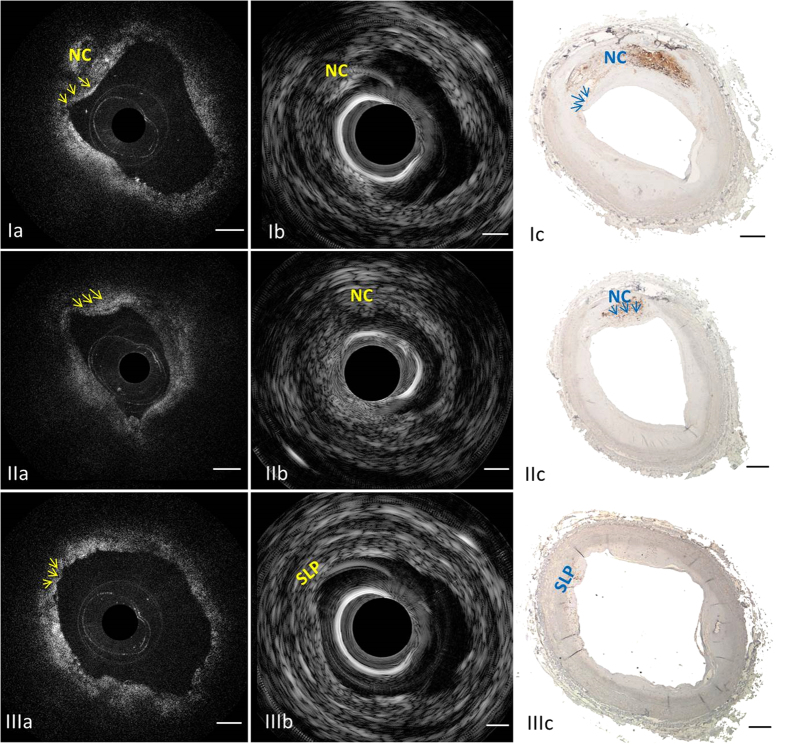
Characterizing human atherosclerotic plaques by the ultrafast IVUS-OCT system. First row: Example of a TCFA. (**Ia**) OCT image in which arrows point at the fibrous cap; (**Ib**) corresponding IVUS image indicates the location of necrotic core; (**Ic**) photo of the corresponding histology slide with CD 68 stain, highlighting macrophages and necrotic core. Middle row: A false positive case of TCFA diagnosis based on IVUS-only (**IIb**) was produced due to the insufficient resolution and sensitivity. Size of the thick cap can be determined by the corresponding OCT (**IIa**) and CD 68 histology (**IIc**). Bottom row: A false positive case of TCFA diagnosis based on OCT-only (**IIIa**) was produced due to OCT’s limited penetration depth. A small lipid pool can be determined by IVUS (**IIIb**) and CD 68 histology (**IIIc**). Arrows denote the fibrous cap. NC: necrotic core; SLP: small lipid pool. Scale bar: 0.5 mm.

To investigate the capability of recognition of vulnerable plaques using this IVUS-OCT technology, we imaged 50 human coronary arteries *in vitro* and characterized the plaques. Examples are shown in Fig. 3. TCFA and false TCFA can be differentiated using IVUS-OCT images (Fig. 3Ia,b,IIa,b,IIIa,b). Corresponding histology photos (Fig. 3Ic,IIc and IIIc), where the positive CD 68 stain regions confirmed the existence of macrophages and necrotic cores, validated the diagnosis based on IVUS-OCT images. Cross-sectional OCT (Fig. 3Ia) and IVUS (Fig. 3Ib) images clearly demonstrate complementary information about the coronary artery wall. OCT allows for recognition of the thin cap feature, which is not seen in the IVUS image, thanks to its high spatial resolution. IVUS detects the presence of an echolucent region, representing the necrotic core (“NC” in Fig. 3Ib). In Fig. 3II, physicians diagnosed this ROI as TCFA when only reading the IVUS image. This misdiagnosis was caused by the insufficient resolution and sensitivity of IVUS. However, using the OCT image as reference (see Fig. 3IIa), a signal low region with a diffuse boundary (i.e., lipid plaque) could be visualized, and the thickness of the signal high region (fibrous cap) could be measured to be greater than 150 μm. In Fig. 3III, physicians diagnosed this ROI as TCFA when only reading the OCT image. Macrophages accumulated in the intimal surface created a TCFA-like image. This misdiagnosis was caused by OCT’s limited penetration depth. However, using the IVUS image as reference (see Fig. 3IIIb), no underlying necrotic core could be found.

## Discussion

In this study, we demonstrated the feasibility of an ultrafast integrated OCT-IVUS system to image and classify atherosclerotic plaques *in vivo*. We confirmed that the full integration of two complementary techniques of OCT and IVUS permits accurate evaluation of total plaque burden and plaque morphology by using an *in vitro* human cadaver study. The fully integrated imaging system demonstrated in this paper is well suited to identify TCFA. This is because it has a high resolution to identify thin cap and, simultaneously, deep penetration to visualize the entire plaque mass. False TCFA (see Fig. 3II and III) can be clearly differentiated from the true vulnerable plaque (see Fig. 3I) when using both IVUS and OCT information. This simultaneous IVUS-OCT imaging technique has many advantages over previously reported reconstruction techniques^9^,^21^, which used separately obtained OCT and IVUS data sets, in evaluating the two characteristics of TCFA. First, the integrated system reduces the complexity of co-registering separately obtained OCT and IVUS data sets. Second, the operation time and cost of procedures will be highly reduced compared to using an IVUS catheter and OCT catheter separately, and requiring placement of the two catheters (IVUS and OCT) in separate steps. Furthermore, under the guidance of IVUS, a minimal dose of OCT flushing agents is needed, which diminishes the side effects caused by such agents. The fully integrated IVUS-OCT technology has great potential to accelerate the clinical development of real-time accurate identification of vulnerable plaques in humans. In our previous study, we developed a 20 fps IVUS-OCT system^18^,^19^. However, the limited speed posed a significant barrier to translate this technology for clinical imaging^21^,^28^. Limited speed means more injection of contrast agents and also a higher risk of catheter-induced spasm^24^. Here, we successfully addressed this challenge by the development and *in vivo* application of a 72 fps IVUS-OCT imaging system and catheter. This integrated system is able to image at ~2.5 times the speed of common commercially available IVUS-only imaging systems. This breakthrough was achieved through a series of technical advancements, including a more advanced IVUS pulser/receiver, electrical slip ring, graphic processing unit, solid state drive, and customized catheter design. This ultrafast system has the capability to perform volumetric imaging of coronary arteries in several seconds and has the potential to evaluate plaque vulnerability with great accuracy^9^,^13^. This work greatly narrows the gap in translating the IVUS-OCT technology to clinical applications.

Current clinical engineered efforts have been focused on improved vulnerable plaque identification and characterization to aid in risk stratification by developing improved diagnostic tools. High definition IVUS was developed and released to the market recently^29^,^30^,^31^. Another modality, near infrared spectroscopy (NIRS), which detects different molecules by its unique absorption properties, is now combined with IVUS and currently under investigation in large-scale clinical studies^32^,^33^. However, neither of these technologies has shown the ability to detect the thin fibrous cap of a vulnerable plaque. Many novel imaging methods, such as OCT-fluorescence imaging, intravascular photoacoustic imaging, resonant acoustic radiation force optical coherence elastography, and laser speckle elastography^34^,^35^,^36^,^37^,^38^, are being developed and may provide new information for physicians regarding vulnerable plaques. However, most of these imaging technologies have only been studied for a limited number of years, and the clinical evidence proving that such image-derived vulnerable plaque is associated with an increased risk of ACS is still imperfect. In addition, other prospective investigations, including those that study long term outcomes, still need to be performed on a large sample of patients before such technology is widely accepted in clinical practice. The lack of clinical data, long term evaluation and standardized criteria may delay the adoption of those technologies. Conversely, there are a great number of clinical trials that have verified the effectiveness and promoted the wide use of OCT and IVUS technologies independently^39^,^40^,^41^. The conclusions from these trials can be reasonably and logically generalized to an integrated IVUS-OCT technology and also direct its development. There were also several studies that proved the combined use of OCT and IVUS can provide powerful complementary information for coronary imaging applications^9^,^13^,^14^,^15^,^16^. Thus, the integrated IVUS-OCT technology is likely to be the most currently accessible and reliable approach to provide accurate identification of vulnerable plaque^9^ in clinical practice and tackle the burgeoning challenge of ACS morbidity and mortality in today’s world.

Last but not least, this research built a solid foundation for other optical-ultrasonic dual modality imaging techniques, such as functional photoacoustic imaging^42^ and acoustic radiation force induced optical electrography^37^. Several described developments in this paper can be applied to other systems to enable ultrafast imaging capability *in vivo*.

## Methods

### CNR evaluation of the ultrafast IVUS sub-system

The tissue-mimicking phantom used for evaluating IVUS image quality is made of 1.9 wt.% agar. 2 wt.% silicon dioxide powders were added as acoustic scatters. Contrast noise ratio of IVUS image is a critical parameter for image quality evaluation. In this study, CNR is calculated through the following equation:


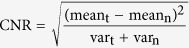


where mean_**t**_ and mean_**n**_ denote the mean of the imaging target and the mean background noise, respectively; var_**t**_ and var_**n**_ represent the signal variance of the imaging target and the background noise, respectively. Imaging target is the agar phantom and the background noise is the water-filled lumen for this study^27^.

### *In vivo* rabbit imaging

This study was conducted according to the guidelines of the National Institutes of Health (NIH) in accordance with the Guide for the Care and Use of Laboratory Animals (ILAR, NAP, Washington, DC, 2011). The Institutional Animal Care and Use Committee at the University of California, Irvine approved the study for the use of New Zealand white rabbits. Animal care and use was performed by qualified individuals and supervised by clinical veterinarians. All facilities and transportation complied with current legal requirements and our animal facilities meet the standards of the American Association for Accreditation of Laboratory Animal Care. Anesthesia was used in all surgical interventions.

The first step was to develop a model of atherosclerosis. Two male New Zealand white rabbits were fed a high-cholesterol diet (0.5% cholesterol, 6% peanut oil, Newco Distributors, Inc.). After 1 week on the diet, de-endothelialization procedures were performed on all rabbits. We inserted a 4F Fogarty arterial catheter via a femoral artery and advanced to the level of diaphragmatic recess of the aorta. Next, we inflated the balloon catheter to 8 atm and pulled back in the abdominal aorta to the level of approximately the common iliac arteries. The balloon was deflated and the above de-endothelialization process was repeated for a total of three passes within the abdominal aorta. Following recovery from the de-endothelialization procedure, rabbits were maintained on the high cholesterol diet throughout the post-procedure period. After 11–12 weeks, lesion formation was mature. It is believed that these lesions produced by balloon de-endothelization and high-cholesterol diet are similar to human atherosclerosis plaques^43^.

In the second phase of the study, we proceeded with imaging the mature atherosclerotic plaques using the IVUS-OCT system. A total of 12 volumetric data sets were obtained from 2 atherosclerotic rabbits and 2 healthy rabbits (control). During the imaging procedures, the rabbits were anesthetized, intubated and mechanically ventilated followed by general anesthesia administration. Under general anesthesia, a laparotomy was performed to expose the abdominal aorta. A 6-F arterial catheter was then inserted into the aorta^44^ at the level of the renal arteries in a superior to inferior direction to correspond to anterograde arterial blood flow towards the lower extremities. The IVUS-OCT catheter was advanced through the 6-F arterial catheter and into the abdominal aorta. About 72 mm long aorta segments were imaged during 4 seconds. Omnipaque (a conventional clinically used CT contrast agent) was used for blood clearance for OCT imaging. Before imaging, a 12 cc flushing agent was flushed into the rabbit aorta at ~3 cc/s. After imaging, the imaged area of each abdominal aorta was dissected, fixed, embedded, sectioned to 6 μm-thick slides and stained with H&E stain.

### Human coronary artery imaging

Over 300 ROI from 25 cadavers were imaged with the IVUS-OCT integrated system in phosphate buffered saline. After imaging, each coronary artery was decalcified, embedded, and sectioned to 6 μm-thick slides. Every 500 μm, two slides were collected and stained for identifying the histological components of the ROI. One sectioned slide was stained with H&E, and the other with immunostain CD 68. Two cardiologists read the corresponding IVUS-OCT image pairs and classified them as TCFA or non-TCFA, blinded to each other’s diagnosis, and histological results. They used OCT images to measure the thickness of the fibrous cap and IVUS images to evaluate the lipid pool size.

## Additional Information

**How to cite this article**: Li, J. *et al.* Ultrafast optical-ultrasonic system and miniaturized catheter for imaging and characterizing atherosclerotic plaques *in vivo*. *Sci. Rep.* **5**, 18406; doi: 10.1038/srep18406 (2015).

## Supplementary Material

Supplementary Information

Supplementary video
