# Supplementary material for: Ultrafast optical-ultrasonic system and miniaturized catheter for imaging and characterizing atherosclerotic plaques in vivo
Source: Sci Rep. 2015 Dec 18;5:18406. doi: 10.1038/srep18406 (PMC4683418; doi:10.1038/srep18406)
Supplement: Supplementary Information [file srep18406-s1.doc]

## Ultrafast optical-ultrasonic system and miniaturized catheter for imaging and characterizing atherosclerotic plaques *in vivo*

##

Jiawen Li^ab*^, Teng Ma^c*^, Dilbahar Mohar^d^, Earl Steward^d^, Mingyue Yu^c^, Zhonglie Piao^a^, Youmin He^a^, K. Kirk Shung^c^, Qifa Zhou^c^, Pranav M. Patel^d^, and Zhongping Chen^ab^

^a^Beckman Laser Institute, University of California, Irvine, 1002 Health Sciences Rd. Irvine, CA 92617, USA, Tel: 949-824-1247, ^b^Department of Biomedical Engineering, University of California, Irvine, Irvine, CA, 92697-2700, USA, Tel: 949-824-1247, Fax: 949-824- 8413; ^c^NIH Ultrasonic Transducer Resource Center, University of Southern California, Los Angeles, CA 90089, Tel: 213-740-9475, Fax: 213- 821-3897; ^d^School of Medicine, University of California, Irvine, 101 The City Drive South, Orange, CA, 92868, USA, Tel: 714-456-6699, Fax: 714-456-8895. * Co-first authors.

**Video Legend**

Supplementary video: Ultrafast IVUS-OCT imaging of a rabbit abdominal aorta *in vivo*. The top movie shows IVUS and corresponding OCT images of a rabbit abdominal aorta, obtained *in vivo* simultaneously at 72 fps. The bottom video shows the three-dimensional rendering of the obtained IVUS-OCT data set. Left, IVUS images and rendering (blue). Right, OCT images and rendering (red).
